# Ten fish mitogenomes of the tribe Gobionini (Cypriniformes: Cyprinidae: Gobioninae)

**DOI:** 10.1080/23802359.2018.1467236

**Published:** 2018-07-27

**Authors:** Yuhuo Li, Kai Cao, Cuizhang Fu

**Affiliations:** Ministry of Education Key Laboratory for Biodiversity Science and Ecological Engineering, Coastal Ecosystems Research Station of the Yangtze River Estuary, Shanghai Institute of Eco-Chongming (SIEC), Fudan University, Shanghai, China

**Keywords:** Gobionini, Gobioninae, Cyprinidae, mitochondrial genome, phylogeny

## Abstract

Freshwater fishes of the subfamily Gobioninae (Cypriniformes: Cyprinidae) are composed of three major clades, the tribes Gobionini and Sarcocheilichthyini, and a *Hemibarbus*–*Squalidus* group. In this study, we determined ten complete fish mitogenomes from eight of all twelve genera in the tribe Gobionini. The ten mitogenomes displayed similar patterns in gene arrangements, codon use and gene overlaps with the length of 16,605–16,617 bp and base compositions slightly A + T bias of 56.1–58.0%. Our phylogeny of the tribe Gobionini revealed that the genera *Gobio*, *Xenophysogobio, Gobiobotia*, *Saurogobio* and *Pseudogobio* were monophyletic, and other genera *Abbottina*, *Biwia*, *Microphysogobio* and *Platysmacheilus* were paraphyletic or polyphyletic. The findings indicate that the genera classifications of Gobionini are needed to be further confirmed.

Freshwater fishes of the subfamily Gobioninae (Cypriniformes: Cyprinidae) can be divided into three major clades, the tribes Gobionini and Sarcocheilichthyini, and *Hemibarbus*–*Squalidus* group (Tang et al. 2011). The tribe Gobionini includes twelve genera, *Abbottina*, *Biwia*, *Gobio*, *Gobiobotia*, *Huigobio*, *Mesogobio*, *Microphysogobio*, *Platysmacheilus*, *Pseudogobio*, *Romanogobio*, *Saurogobio* and *Xenophysogobio* (Tang et al. 2011). In this study, we determined complete mitogenomes of ten species of fishes from eight genera in the Gobionini.

Ten sampled specimens of Gobioninae fishes were preserved in 95% ethanol and deposited in the Zoological Museum of Fudan University (FDZM), China. These fishes were collected in China along with *Abbottina rivularis* (voucher FDZM-ARZLT20110701) from Zhalantun City (48.02°N, 122.73°E), *A. lalinensis* (FDZM-ALQY20110401) from Qingyuan Manchu Autonomous County (42.11°N, 124.97°E), *Gobio cynocephalus* (FDZM-GCJAL20110701) from Jalaid Banner (46.78°N, 122.68°E), *Gobiobotia pappenheimi* (FDZM-GPTL20110501) from Tieling City (42.29°N, 123.84°E), *Pseudogobio vaillanti* (FDZM-PVKD20100701) from Kuandian Manchu Autonomous County (40.75°N, 124.77°E), *Xenophysogobio nudicorpa* (FDZM-XNJJ20130501) and *X. boulengeri* (FDZM-XBJJ20130501) from Jiangjin District (29.29°N, 106.26°E), *Saurogobio dabryi* (FDZM-SDXS20120401) from Xiushui County (29.03°N, 114.57°E), *Platysmacheilus longibarbatus* (FDZM-PLND20111101) and *Huigobio chenhsienensis* (FDZM-HCND20111101) from Ningdu County (26.46°N, 116.00°E). Total genomic DNA was extracted from muscle tissue using high salt method (Miller et al. 1988). Mitogenomes were sequenced using Sanger sequencing and assembled using *Pseudorasbora elongata* (Chen et al. 2015) as reference.

The lengths of ten new mitogenomes (GenBank accession numbers: KU314691–KU314700) varied from 16,605 to 16,617 bp, with base compositions slightly A + T bias of 56.1–58.0%. These mitogenomes were composed of 13 protein-coding genes, 22 transfer RNA (tRNA) genes, 2 ribosomal RNA (rRNA) genes, and 1 control region. They displayed similar patterns in gene arrangements, codon use and gene overlaps, which have also been reported in other Gobioninae mitogenomes (Hwang et al. 2013; Chen et al. 2015). All genes were encoded on the heavy strand, except for *ND6* and 8 tRNA genes [*-Gln*, *-Ala*, *-Asn*, *-Cys*, *-Tyr*, *-Ser* (UCN), *-Glu* and *-Pro*]. Two types of start codons (ATG and GTG) and three types of stop codons (TAA, TAG and T–) were used in protein-coding genes for each of the ten mitogenomes except for the mitogenome of *P. longibarbatus,* which used TGA as stop codon in its *COX1* gene. Four pairs of adjacent protein-coding genes, *ATP8–ATP6*, *ATP6*–*COIII*, *ND4L–ND4*, and *ND5–ND6* had the overlapped size of 7, 1, 7, and 4 bp, respectively.

Phylogenetic relationship of fishes in the Gobionini (Figure 1) was reconstructed based on 38 mitogenomes in Mrbayes ver. 3.2.6 (Ronquist et al. 2012) and Raxml ver. 8.2.10 (Stamatakis 2014) using six partitions, i.e. each codon of all protein-coding genes (excluding *ND6*), 12S rRNA gene, 16S rRNA gene, and all tRNA genes. Outgroup taxa were selected based on a previous study (Tang et al. 2011). Our phylogeny revealed that (i) the tribe Gobionini was a monophyletic group, and (ii) the genera *Gobio, Xenophysogobio, Gobiobotia, Saurogobio* and *Pseudogobio* were monophyletic, and other genera *Abbottina, Biwia, Microphysogobio* and *Platysmacheilus* were paraphyletic or polyphyletic. The findings indicate that the genera classifications of Gobionini are needed to be further confirmed.

**Figure 1.**
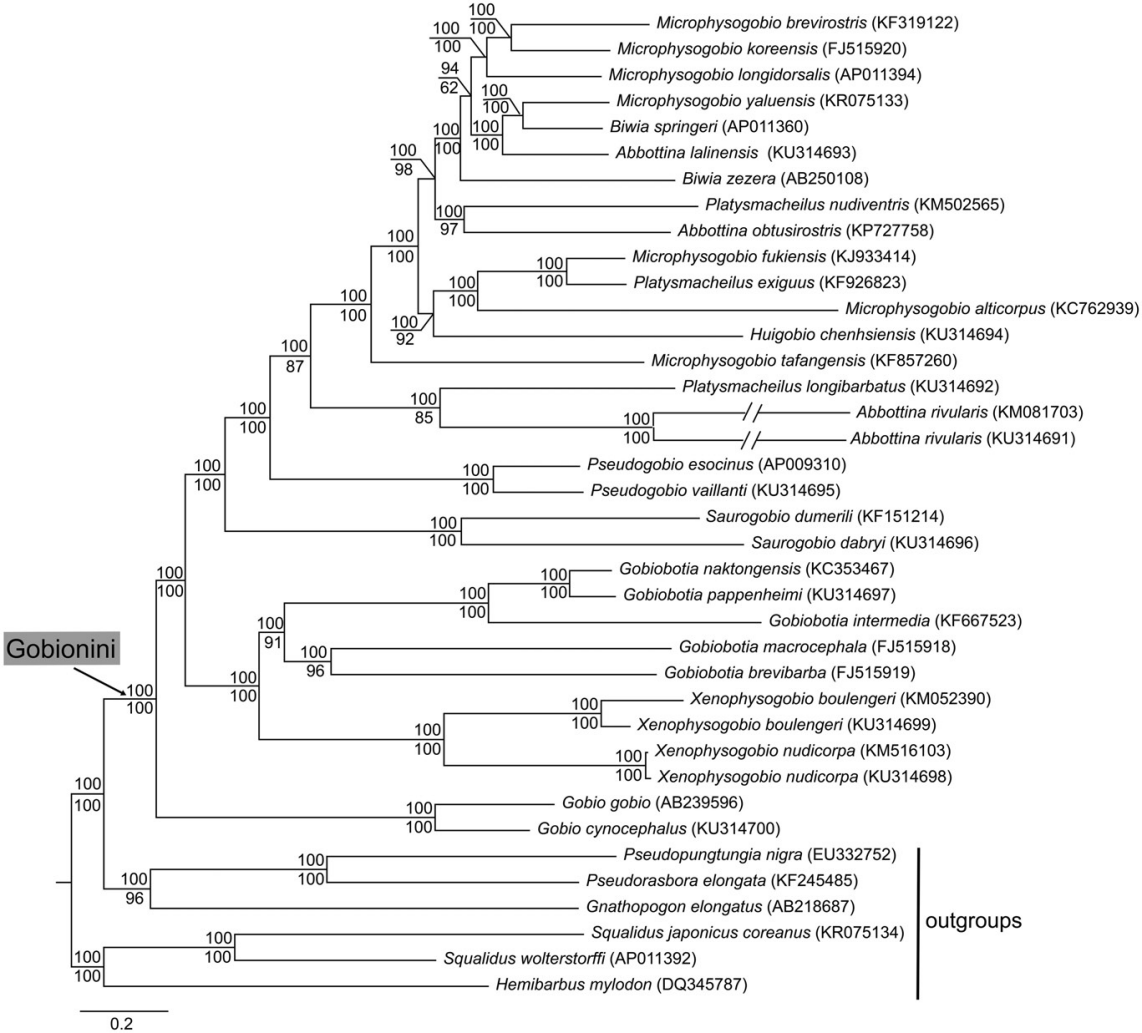
Topology of Bayesian tree for 29 fish species in the tribe Gobionini and 6 outgroup species based on mitogenome sequences. Bayesian posterior probabilities are shown above branches for Bayesian analyses and bootstrap confidences (1000 replicates) are shown below branches for maximum likelihood analyses. GenBank accession numbers are given in parentheses.
